# The Association between Maternal–Fetal Medicine Physician Density and Pregnancy Outcomes

**DOI:** 10.1055/a-2717-3951

**Published:** 2025-10-16

**Authors:** Tetsuya Kawakita, Rula Atwani, Misa Hayasaka, Lindsay Robbins, George Saade

**Affiliations:** 1 EVMS Department of Obstetrics and Gynecology, Macon and Joan Brock Virginia Health Sciences at Old Dominion University, Norfolk, Virginia, United States; 2 EVMS Center for Maternal and Child Health Equity and Advocacy, Macon and Joan Brock Virginia Health Sciences at ODU, Norfolk, Virginia, United States

**Keywords:** maternal death, maternal mortality, physician density, pregnancy-related mortality

## Abstract

**Objective:**

We sought to examine the association between maternal–fetal medicine (MFM) physician density and adverse pregnancy outcomes at the state level.

**Study Design:**

This was a cross-sectional analysis of publicly available, state-level data from 2018 to 2021, including Natality, Multiple Cause-of-Death, and Fetal Death databases. The number of active MFM physicians per year was obtained for each state from the American Medical Association Masterfile. The primary exposure was the density of MFM per state, categorized into three groups: (1) low density (<30 MFM physicians per 100,000 live births), (2) moderate density (30–59 MFM physicians per 100,000 live births), and (3) high density (≥60 MFM physicians per 100,000 live births). Our primary outcome was maternal mortality during pregnancy and up to 42 days postdelivery. Our secondary outcomes were pregnancy-related mortality up to 365 days postdelivery and stillbirth. We calculated adjusted incident rate ratios (aIRR) and average marginal effect (AME) with 95% confidence intervals (95% CI) using multivariable negative binomial mixed effects regression models.

**Results:**

Overall, there were 14,792,743 live births, 3,440 maternal mortalities, 4,980 pregnancy-related mortalities, and 90,848 stillbirths included. The median MFM density across states was 31.6 per 100,000 live births (interquartile range: 21.9–42.5). States with high MFM density had a reduced risk of maternal mortality (aIRR: 0.70; 95% CI: 0.58–0.85) and pregnancy-related mortality (aIRR: 0.83; 95% CI: 0.71–0.98) compared with states with low MFM density, corresponding to 7.29 (AME: 95% CI: 3.58–11.00) and 5.57 (AME: 95% CI: 0.74–10.40) less mortality per 100,000 live births, respectively. States with moderate MFM density had a similar risk of maternal mortality compared with low MFM density states (aIRR: 1.02; 95% CI: 0.87–1.20).

**Conclusion:**

High MFM-density states have a decreased risk of maternal mortality compared with low MFM-density states, suggesting a critical role of MFM in reducing maternal mortality.

**Key Points:**

- An association between MFM physician density and maternal mortality is unclear.
- States with higher MFM density had a lower risk of maternal mortality than those with fewer.
- This study highlights the importance of increasing MFM access in underserved areas to potentially reduce maternal mortality.

## Introduction

The maternal mortality rate in the United States has been on the rise, increasing from 17.4 per 100,000 live births in 2018 to 32.9 per 100,000 live births in 2021. 1 The major contributors to maternal deaths—accounting for nearly 75% of cases—are severe maternal morbidity, which includes conditions such as hemorrhage, infections, hypertensive disorders, and complications arising from unsafe abortions. 2 It is estimated that 20 to 40% of maternal morbidity and mortality are preventable with timely and appropriate interventions. 3

While obstetricians and gynecologists (OBGYNs) are vital for routine maternal care, maternal–fetal medicine (MFM) physicians offer a more advanced level of care for complicated pregnancies, providing clinical care that can be lifesaving. As specialists trained in managing high-risk pregnancies, MFM physicians are uniquely positioned to identify and treat complications that can arise during pregnancy, labor, and postpartum periods. However, access to MFM physicians is unevenly distributed across the United States, with the majority practicing in urban areas. 4 Although there are approximately 140 MFM fellowship positions available each year, 5 most of these training programs are concentrated in suburban settings. This geographic imbalance raises concerns, particularly for rural and underserved areas, where lower densities of MFM physicians may contribute to higher rates of severe maternal morbidity.

A study published two decades ago suggested that increasing the number of MFM physicians by 5 per 10,000 live births could lead to a 27% reduction in maternal mortality risk. 6 As maternal mortality rates continue to rise, there is an urgent need to assess the distribution and impact of MFM physicians on maternal health outcomes. This study aims to explore the association between MFM physician availability and pregnancy outcomes, focusing on how their presence—or lack thereof—affects maternal mortality.

## Materials and Methods

This was a cross-sectional analysis of publicly available, state-level data from the Centers for Disease Control and Prevention, Wide-Ranging Online Data for Epidemiologic Research (CDC WONDER) database, covering the period from January 2018 to December 2021. 7 The CDC WONDER includes comprehensive databases, including Natality, Underlying Cause-of-Death, and Fetal Death databases. Data on live births were sourced from the Natality databases, providing counts of live births occurring in the United States (50 states and the District of Columbia) to U.S. residents. 8 Mortality data were retrieved from the Underlying Cause-of-Death databases, based on U.S. resident death certificates, each listing a single underlying cause of death, up to 20 additional multiple causes, and demographic information. 9 Stillbirth data were drawn from the Fetal Death databases, which include fetal deaths at 20 weeks of gestation or more within the United States. 10 The analysis was limited to individuals aged 15 to 44 years, as prior studies indicate a high false-positive rate for maternal mortality in those aged 45 years and older. 11 Consistent inclusion and exclusion criteria were applied across datasets. Institutional Review Board review was deemed unnecessary, as only publicly available de-identified data were used. This study followed the Strengthening the Reporting of Observational Studies in Epidemiology reporting guideline.

## Exposures

We obtained de-identified data on practicing MFM physicians from the American Medical Association Masterfile for 2018 to 2021. This dataset provided yearly counts of active MFM physicians per state. To ensure accuracy, MFM fellows in training and retired MFM physicians were excluded, focusing exclusively on actively practicing physicians.

To calculate MFM physician density, we divided the number of active MFM physicians in each state by the total number of live births for the corresponding year and state, scaling the result per 100,000 live births to standardize comparisons across states and years. States were categorized annually into three groups based on MFM density median across states of 31 MFM physicians per 100,000 live births: (1) low density (<30 MFM physicians per 100,000 live births), (2) moderate density (30–59 MFM physicians per 100,000 live births), and (3) high density (≥60 MFM physicians per 100,000 live births). These thresholds were chosen to reflect meaningful differences in access to MFM care across states, with < 30 as low access, 30 to 59 as moderate access consistent with the national median, and ≥60 as high access, capturing states with comparatively high MFM physician availability.

## Outcomes

The primary outcome was maternal mortality. Maternal death is defined by the World Health Organization as “the death of a woman during pregnancy and up to 42 days postdelivery, regardless of the pregnancy's duration or location, from any cause related to or aggravated by the pregnancy or its management, excluding accidental or incidental causes.” 12 Therefore, cases with an underlying cause of death assigned to the International Statistical Classification of Diseases, 10th Revision (ICD-10) code numbers A34, O00–O95, and O98–O99 were included. 13 Secondary outcomes included pregnancy-related mortality and stillbirths. The pregnancy-related mortality rate, which includes late maternal deaths (within 1 year postdelivery, coded as ICD-10 O96 and O97), was examined due to elevated risks within the first postpartum year. 14 Stillbirth was defined as fetal death at 20 weeks of gestation or later. All outcomes were assigned based on the state where delivery occurred.

## Data Analysis

We plotted the number of live births, maternal mortality, pregnancy-related mortality, stillbirths, and MFM by calendar year. The annual trend was statistically examined using Poisson regression. We integrated data from 2018 to 2021 and applied a Bayesian spatial autoregressive model using the Geostan package in R to account for spatial dependencies. This Bayesian spatial analysis allowed us to estimate adjusted state-wise averages of MFM density and maternal mortality rate and visualize the results on a state-level map. 15 16

We examined individual characteristics by MFM density. To address clustering within states, we employed multivariable negative binomial mixed effects models with Huber–White robust estimates, calculating adjusted incident rate ratios (aIRRs) with 95% confidence intervals (CIs) for maternal mortality, pregnancy-associated mortality, and stillbirths, using low MFM density as the reference. The negative binomial regression model was chosen due to overdispersion. Models were adjusted for state-specific demographics (proportion of non-White individuals, obesity, chronic hypertension, pregestational diabetes, and education levels below high school) from the Natality databases, as well as state-specific poverty rates from the United States Census Bureau. 17 In addition, the year variable was adjusted to account for annual variation and the spike in maternal mortality during the coronavirus disease 2019 (COVID-19) pandemic. Lastly, we also adjusted for the density of OBGYNs and midwives per 100,000 births. These covariates were selected based on established associations with maternal mortality in the literature. 18 19 20 21 22 Additionally, to improve the interpretability, adjusted rates and average marginal effects (AME) were calculated using the marginal standardization form of predictive margins; we estimated the probability in each group by fixing state-level characteristics at the average level of the categories. The AME represents the difference in adjusted rates across states, helping to mitigate potential misinterpretation, especially when baseline risks are low. 23 Furthermore, we plotted the adjusted rates of maternal mortality per 100,000 live births against the MFM density.

## Sensitivity Analyses

To ensure robustness, several sensitivity analyses were performed. First, a leave-one-out analysis was conducted, sequentially excluding each state to examine any disproportionate impact on results. Second, to account for the potential influence of antenatal MFM care, analyses were repeated using the state of residence rather than the delivery location. Third, stability across classifications was evaluated using alternative MFM density categories (<20, 20–39, 40–59, and ≥ 60 MFM physicians per 100,000 live births). Fourth, we calculated the E-value to estimate the minimum strength of association required from an unmeasured confounder to explain the observed relationship between MFM density and MMR. 24

Effects were considered significant if the 95% CI did not include the null or if the *p* -value was less than 0.05. All statistical analyses were conducted using Stata version 18.5 (StataCorp, College Station, Texas, United States) and R version 4.4.1 (R Foundation for Statistical Computing, Vienna, Austria).

## Results

Overall, there were 14,792,743 live births, 3,440 maternal mortalities, 4,980 pregnancy-related mortalities, and 90,848 stillbirths during our study period from January 2018 to December 2021. The median MFM density across states was 31.6 per 100,000 live births (interquartile range: 21.9–42.5).

**Fig. 1:**
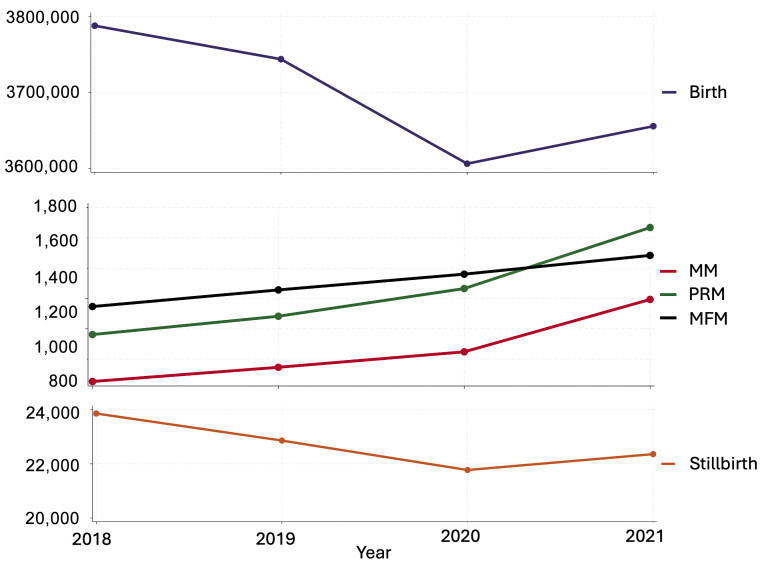
Trends in births, maternal outcomes, maternal–fetal medicine physicians, and stillbirths (2018–2021). The blue line represents the trend in the number of births, the red line represents the trend in the number of maternal morbidity and mortality, the green line represents the trend in the number of pregnancy-related mortality, the black line represents the trend in the number of MFM physicians, and the orange line represents the trend in the number of stillbirths. Each dot on the lines represents the observed yearly value for each metric. MFM, maternal–fetal medicine; MM, maternal mortality; PRM, pregnancy-related mortality.

Fig. 1 presents the trends in the number of births, maternal outcomes, MFM physicians, and stillbirths from 2018 to 2021 in the United States. During this period, the number of live births significantly decreased ( *p* < 0.001), while the number of maternal mortality, pregnancy-related mortality, and MFM physicians increased (all *p* < 0.001). There was no clear trend for stillbirth ( *p* = 0.07).

**Fig. 2:**
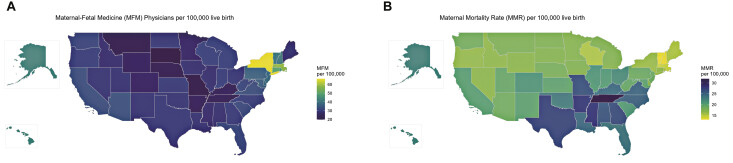
Distribution of MFM physicians and maternal mortality per 100,000 live births across states. ( **A** ) Displays the density of MFM physicians per 100,000 live births across states. States are color-coded on a gradient from purple (lower density) to yellow (higher density), with yellow indicating areas with the highest density of MFM physicians. ( **B** ) Displays maternal mortality rates (MMR) per 100,000 live births across states. States are color-coded on a gradient from yellow (lower MMR) to green and purple (higher MMR), with purple highlighting areas with the highest maternal mortality rates. MFM, maternal–fetal medicine; MMR, maternal mortality rate.

Fig. 2 illustrates the distribution of MFM physician densities and maternal mortality rates across states. The Northeast exhibited the highest density of MFM physicians, with states like Massachusetts and New York having over 50 MFM physicians per 100,000 live births, correlating with lower maternal mortality in 100,000 live births in these regions. In contrast, the South and Midwest showed a comparatively lower density of MFM physicians and higher maternal mortality in 100,000 live births, highlighting disparities in healthcare resources and outcomes across regions.

Demographics are presented in eTable 1 (available in the online version). Individuals in high MFM density states compared with those in other states were more likely to be older, White, less likely to have an education less than a high school degree, and more likely to have chronic hypertension. Furthermore, high MFM density states were more likely to have a higher number of OBGYNs and midwives.

**Fig. 3:**
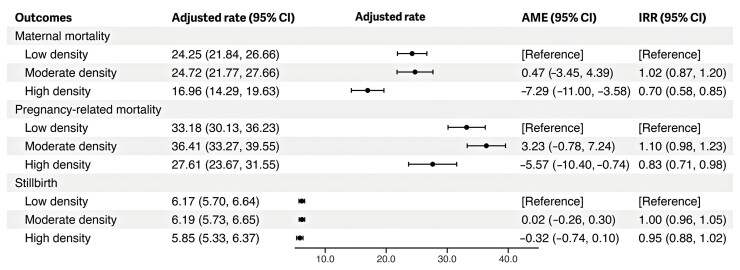
Maternal mortality, pregnancy-related mortality, and stillbirth, stratified by MFM physician density levels. Each dot represents the adjusted rate for a specific outcome at a given density level. The horizontal line extending from each dot shows the 95% confidence interval for that estimate. Models were adjusted for state-specific rates of non-White individuals, chronic hypertension, pregestational diabetes, education levels below high school, poverty rates, OBGYN/Midwives density, and year. AME, average marginal effect; CI, confidence interval; IRR, incident rate ratio.

Maternal mortality, pregnancy-related mortality, and stillbirth rates according to MFM density are displayed in Fig. 3 . States with high MFM density had a reduced risk of maternal mortality (aIRR: 0.70; 95% CI: 0.58–0.85) and pregnancy-related mortality (aIRR: 0.83; 95% CI: 0.71–0.98) compared with states with low MFM density, corresponding to 7.29 (AME: 95% CI: 3.58–11.00) and 5.57 (AME: 95% CI: 0.74–10.40) less mortality per 100,000 live births, respectively. States with moderate MFM density had a similar risk of maternal mortality compared with low MFM density states (aIRR: 1.02; 95% CI: 0.87–1.20). Unadjusted rates and IRRs were consistent with the primary analysis ( eTable 2 , available in the online version). In contrast, stillbirth rates did not differ significantly across MFM density groups.

**Fig. 4:**
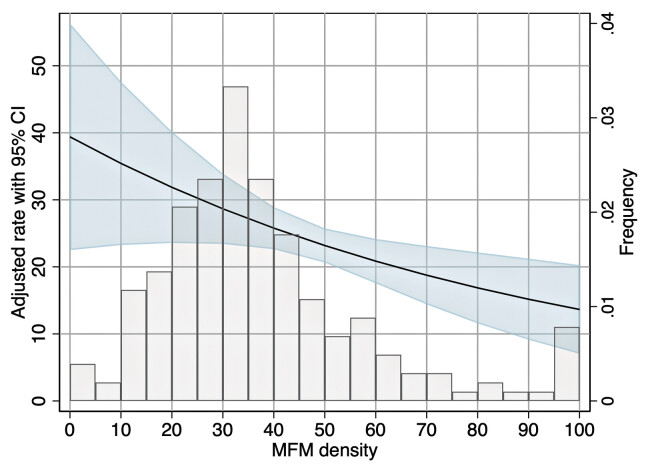
Frequency distribution of MFM density and its association with maternal mortality. Gray bars represent the frequency of states by MFM physician density per 100,000 live births. The black line represents the adjusted rate for maternal mortality across MFM density levels, with the blue shaded area indicating the 95% confidence interval. Models were adjusted for state-specific rates of non-White individuals, chronic hypertension, pregestational diabetes, education levels below high school, poverty rates, OBGYN/Midwives density, and year. MFM, maternal–fetal medicine.

Fig. 4 depicts the frequency distribution of MFM density and the association between varying MFM density and maternal mortality. As MFM density increased, adjusted rates of maternal mortality decreased.

Sensitivity analyses supported the primary findings. In the leave-one-out analysis, the exclusion of any single state did not affect the observed findings ( eFig. 1 , available in the online version). Analyses based on the state of residence also produced results consistent with the primary analysis ( eFig. 2 , available in the online version). Alternative threshold analyses showed similar patterns, except for the loss of statistical significance in the association between MFM density and pregnancy-related mortality ( eFig. 3 , available in the online version). Lastly, the E-value for maternal mortality risk in high MFM density states was 2.21, suggesting that any unmeasured confounder would need to have a risk increase of over twofold to fully account for the observed association between high MFM density and reduced maternal mortality.

## Discussion

In this cross-sectional study using national U.S. databases, we found that states with high MFM density had a significantly lower risk of maternal mortality compared with states with low MFM density. In contrast, stillbirth rates showed no significant association with MFM density. Our analysis also highlighted a geographically uneven distribution of MFM physicians, corresponding with disparities in maternal mortality across regions. We found that an MFM density of 60 or more per 100,000 live births is associated with a decreased risk of maternal mortality compared with lower densities. These results remained consistent across multiple sensitivity analyses, underscoring the robustness of our findings.

Nearly two decades ago, a study reported a maternal mortality rate of 7.5 per 100,000 live births, estimating that an additional 50 MFM physicians per 100,000 live births could reduce maternal mortality by 27%. 6 By comparison, the current maternal mortality rate has risen sharply to 32.9 per 100,000 live births in 2021—over four times higher than previously reported. 18 While this increase could partly be explained by changes in maternal mortality surveillance methodologies, such as the introduction of the “pregnancy checkbox,” 13 other factors, including rising maternal age, higher obesity rates, and a growing prevalence of preexisting medical conditions, likely play a role. 25 26 Notably, our study did not use the “pregnancy checkbox” for mortality classification to reduce potential misclassification bias.

This study highlights the critical role of MFM density in improving maternal health outcomes. Our findings underscore the importance of ensuring adequate distribution of MFM physicians, particularly in regions with elevated maternal mortality rates. Addressing these disparities by allocating MFM resources more evenly across states could improve maternal outcomes, especially in underserved regions with high maternal mortality rates, such as the South and Midwest. Additionally, increased support for MFM training programs in underserved regions, alongside incentives to retain physicians in these areas, could serve as practical steps toward improving access to specialized maternal care. This focus on equitable access to MFM resources aligns with broader public health goals of reducing maternal mortality and achieving health equity across the United States.

MFM physicians can play a critical role in reducing MMR by providing prevention and acute treatment. MFM physicians specialize in the early identification and management of conditions such as hypertension, diabetes, and maternal cardiac diseases, which are closely linked to maternal mortality. 27 By recognizing these risks early, MFMs could implement preventive measures that reduce the likelihood of severe complications.

In addition, MFMs facilitate multidisciplinary management, working alongside specialists such as cardiologists, anesthesiologists, and neonatologists to ensure comprehensive care. MFMs also play a pivotal role in obstetric emergencies such as hemorrhage, severe hypertension, and thromboembolism. 28 Furthermore, MFM physicians help develop clinical care protocols for high-risk patients as well as participate in quality improvement processes, which ultimately improve maternal care standards and outcomes. In underserved areas, they may provide training and consultative support to general obstetricians, indirectly raising the level of care even where MFM density is lower.

Our findings suggest significant research implications. While we observed an association between high MFM density and reduced maternal mortality rate, the specific mechanisms driving this relationship remain unclear. Future studies should explore whether the observed benefits stem from increased access to specialized prenatal care, enhanced management of high-risk pregnancies, or improved interdisciplinary collaboration. Recent trends indicate that one-third of OBGYNs and MFMs relocated within a decade, often to more urban, affluent areas. 4 29 Addressing MFM maldistribution may, therefore, require policy changes or economic incentives, warranting further investigation into strategies for increasing MFM densities in underserved areas.

Our study has several strengths. First, it utilized large, national datasets, offering a broad perspective on MFM distribution and maternal outcomes across diverse U.S. regions, enhancing the generalizability of our results. Second, by examining geographic disparities, the study reveals regional patterns in MFM density and MMR, highlighting areas of healthcare inequity. Third, multiple sensitivity analyses were conducted to ensure consistency across various thresholds and exclusions, reinforcing the robustness of our findings. Lastly, the use of government-provided datasets enhanced data accuracy, reliability, and replicability, enabling transparent analysis on a representative scale.

Our study is not without limitations. As a cross-sectional analysis, it limits causal inferences regarding the relationship between MFM density and maternal mortality. Although robust, our analysis may still be influenced by unmeasured confounders, such as healthcare infrastructure and socioeconomic factors. However, given that the E-value exceeded 2.0 for maternal mortality, it is unlikely that unmeasured confounders alone would account for the observed association. Finally, despite efforts to minimize misclassification by restricting maternal age to 15 to 45 years and excluding the “pregnancy checkbox,” some misclassification risk persists. 11 13 Finally, we were not able to determine the type of individual MFM practices (e.g., solely consultative vs. assuming primary patient care). This lack of detail limits our ability to differentiate MFM roles, which is essential for understanding the direct impact of MFM density on maternal outcomes.

In conclusion, we found a significant association between high MFM density and decreased maternal mortality, highlighting disparities in MFM distribution across the United States. These findings underscore the potential benefits of optimizing MFM density to improve maternal health outcomes, particularly in underserved regions. This study contributes valuable insights into how access to specialized maternal care impacts health disparities.
