# Supplementary material for: The Association between Maternal–Fetal Medicine Physician Density and Pregnancy Outcomes
Source: Am J Perinatol. 2025 Oct 16;43(9):1121–6. doi: 10.1055/a-2717-3951 (PMC13286091; doi:10.1055/a-2717-3951)
Supplement: Supplementary file 1 — Supplementary Material [file 10-1055-a-2717-3951_27241811.pdf]

Online-Only Supplements

The Association between Maternal-Fetal Medicine Physician Density and Pregnancy Outcomes
Tetsuya KAWAKITA, MD; Rula ATWANI, MD; Misa HAYASAKA, MD; Lindsay Robbins, MD, MPH; George SAADE, MD

eTable 1. Demographics

eTable 2. Crude proportions and incident rate ratios.

eFigure 1. Leave-one-out analysis

eFigure 2. Association between MFM density and pregnancy outcomes based on the state of residence

eFigure 3. Association between MFM density using different thresholds and pregnancy outcomes

American Journal of Perinatology | DOI 10.1055/a-2717-3951 | © The Author(s).

eTable 1. Demographics

	All	Low MFM density	Moderate MFM density	High MFM density
n	14792743	6050285	7585900	1156558
Maternal age	29.1 (± 5.7)	28.8 (± 5.8)	29.3 (± 5.7)	30.4 (± 5.6)
Age 35 or greater	2784955 (18.8)	1049475 (17.3)	1450325 (19.1)	285155 (24.7)
Race/ethnicity				
White	7584170 (51.3)	3007712 (49.7)	3936431 (51.9)	640027 (55.3)
Black	2140061 (14.5)	963429 (15.9)	1013327 (13.4)	163305 (14.1)
American Indian	110140 (0.7)	54758 (0.9)	53883 (0.7)	1499 (0.1)
Asian	908757 (6.1)	331263 (5.5)	490365 (6.5)	87129 (7.5)
NHOPI	38285 (0.3)	15813 (0.3)	22140 (0.3)	332 (0.0)
More than one race	338330 (2.3)	130312 (2.2)	185124 (2.4)	22894 (2.0)
Hispanic	3537147 (23.9)	1501194 (24.8)	1804614 (23.8)	231339 (20.0)
Unknown	135853 (0.9)	45804 (0.8)	80016 (1.1)	10033 (0.9)
Education less than high school	1741400 (11.8)	743542 (12.3)	872778 (11.5)	125080 (10.8)
Chronic hypertension	350073 (2.4)	137452 (2.3)	180734 (2.4)	31887 (2.8)
Pregestational diabetes	149249 (1.0)	59202 (1.0)	78074 (1.0)	11973 (1.0)
OBGYN and midwives per 100,000 births	1287.9 (1069.9,1453.6)	1200.4 (928.9,1303.5)	1335.8 (1167.6,1501.2)	1832.8 (1815.4,2041.9)

Abbreviations: MFM (maternal-fetal medicine); NHOPI (Native Hawaiian or Other Pacific Islander); OBGYN (obstetricians and gynecologists)
Numbers are shown as n (%) or mean (± standard deviation).
These numbers are obtained from the Natality databases.

American Journal of Perinatology | DOI 10.1055/a-2717-3951 | © The Author(s).

eTable 2. Crude proportions and incident rate ratios.

Outcomes	Unadjusted rate	cIRR (95% CI)
**Maternal mortality**		
Low density	1457/6050285 (24.1)	[Reference]
Moderate density	1767/7585900 (23.3)	0.99 (0.84, 1.16)
High density	216/1156558 (18.7)	0.71 (0.56, 0.90)
**Pregnancy-related mortality**		
Low density	2010/6050285 (33.2)	[Reference]
Moderate density	2621/7585900 (34.6)	1.01 (0.88, 1.17)
High density	349/1156558 (30.2)	0.75 (0.59, 0.96)
**Stillbirth**		
Low density	38568/6050285 (6.4)	[Reference]
Moderate density	44990/7585900 (5.9)	0.98 (0.90, 1.07)
High density	7290/1156558 (6.3)	1.01 (0.87, 1.17)

Abbreviations: CI (confidence interval); cIRR (crude incident rate ratio)
Rates are shown in 100,000 live births for mortalities and 1,000 live births for stillbirths.

American Journal of Perinatology | DOI 10.1055/a-2717-3951 | © The Author(s).

eFigure 1. Leave-one-out analysis

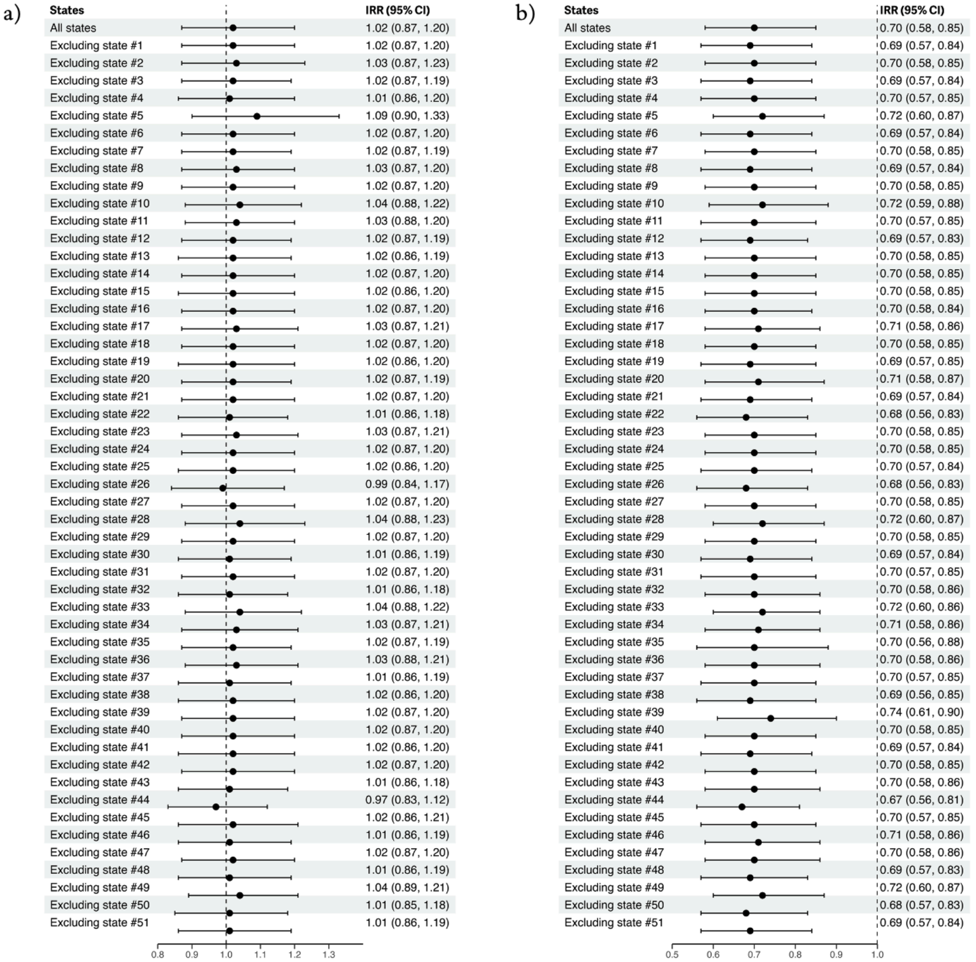

Forest plots of the incident rate ratios (IRRs) with 95% confidence intervals (CIs) for maternal mortality, excluding one state at a time. Each dot indicates the IRR with horizontal lines representing the 95% CI. eFigure 1a represents the comparison between low and moderate MFM density states. eFigure 1b represents the comparison between low and high MFM density states. The dashed vertical line represents an IRR of 1 and serves as the reference line. Models were adjusted for state-specific rates of non-White individuals, chronic hypertension, pregestational diabetes, education levels below high school, poverty rates, OBGYN/Midwives density, and year.

State number: 1. AL, 2. AK, 3. AZ, 4. AR, 5. CA, 6. CO, 7. CT, 8. DE, 9. DC, 10. FL, 11. GA, 12. HI, 13. ID, 14. IL, 15. IN, 16. IA, 17. KS, 18. KY, 19. LA, 20. ME, 21. MD, 22. MA, 23. MI, 24. MN, 25. MS, 26. MO, 27. MT, 28. NE, 29. NV, 30. NH, 31. NJ, 32. NM, 33. NY, 34. NC, 35. ND, 36. OH, 37. OK, 38. OR, 39. PA, 40. RI, 41. SC, 42. SD, 43. TN, 44. TX, 45. UT, 46. VT, 47. VA, 48. WA, 49. WV, 50. WI, 51. WY.

American Journal of Perinatology | DOI 10.1055/a-2717-3951 | © The Author(s).

eFigure 2. Association between MFM density and pregnancy outcomes based on the state of residence

Outcomes	Adjusted rate (95% CI)	Adjusted rate	AME (95% CI)	IRR (95% CI)
Maternal mortality				
Low density	24.46 (22.04, 26.88)		[Reference]	[Reference]
Moderate density	24.74 (22.01, 27.46)		0.27 (-3.49, 4.04)	1.01 (0.87, 1.18)
High density	17.20 (14.51, 19.89)		-7.26 (-11.20, -3.33)	0.70 (0.58, 0.86)
Pregnancy-related mortality				
Low density	33.48 (30.29, 36.67)		[Reference]	[Reference]
Moderate density	36.63 (33.58, 39.67)		3.14 (-1.08, 7.37)	1.09 (0.97, 1.23)
High density	27.85 (23.77, 31.93)		-5.63 (-11.00, -0.27)	0.83 (0.69, 0.99)
Stillbirth				
Low density	6.19 (5.72, 6.66)		[Reference]	[Reference]
Moderate density	6.05 (5.64, 6.46)		-0.14 (-0.43, 0.15)	0.98 (0.93, 1.02)
High density	5.70 (5.24, 6.15)		-0.49 (-0.89, -0.10)	0.92 (0.86, 0.98)

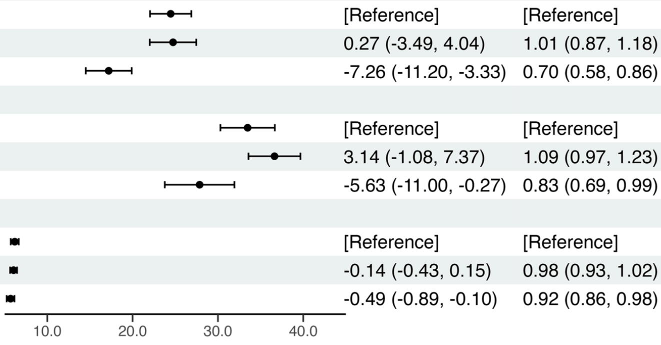

Abbreviations: AME (average marginal effect); CI (confidence interval); IRR (incident rate ratio)
Each dot represents the adjusted rate for a specific outcome at a given density level. The horizontal line extending from each dot shows the 95% confidence interval for that estimate. Models were adjusted for state-specific rates of non-White individuals, chronic hypertension, pregestational diabetes, education levels below high school, poverty rates, OBGYN/Midwives density, and year.

American Journal of Perinatology | DOI 10.1055/a-2717-3951 | © The Author(s).

eFigure 3. Association between MFM density using different thresholds and pregnancy outcomes

Outcomes	Adjusted rate (95% CI)	AME (95% CI)	IRR (95% CI)
Maternal mortality			
<20	23.95 (19.45, 28.46)	[Reference]	[Reference]
20-39	24.08 (21.67, 26.50)	0.13 (-4.37, 4.63)	1.01 (0.83, 1.21)
40-59	26.10 (23.08, 29.12)	2.15 (-3.73, 8.03)	1.09 (0.86, 1.38)
60 or greater	17.42 (14.47, 20.38)	-6.53 (-12.53, -0.53)	0.73 (0.55, 0.97)
Pregnancy-related mortality			
<20	33.99 (28.60, 39.38)	[Reference]	[Reference]
20-39	34.29 (31.63, 36.94)	0.29 (-5.00, 5.58)	1.01 (0.86, 1.18)
40-59	37.34 (33.81, 40.87)	3.35 (-3.29, 9.99)	1.10 (0.91, 1.33)
60 or greater	27.79 (23.65, 31.94)	-6.20 (-13.33, 0.93)	0.82 (0.65, 1.03)
Stillbirth			
<20	6.05 (5.62, 6.47)	[Reference]	[Reference]
20-39	6.17 (5.69, 6.65)	0.12 (-0.29, 0.53)	1.02 (0.95, 1.09)
40-59	6.27 (5.71, 6.82)	0.22 (-0.25, 0.69)	1.04 (0.96, 1.12)
60 or greater	6.00 (5.36, 6.63)	-0.05 (-0.61, 0.52)	0.99 (0.90, 1.09)

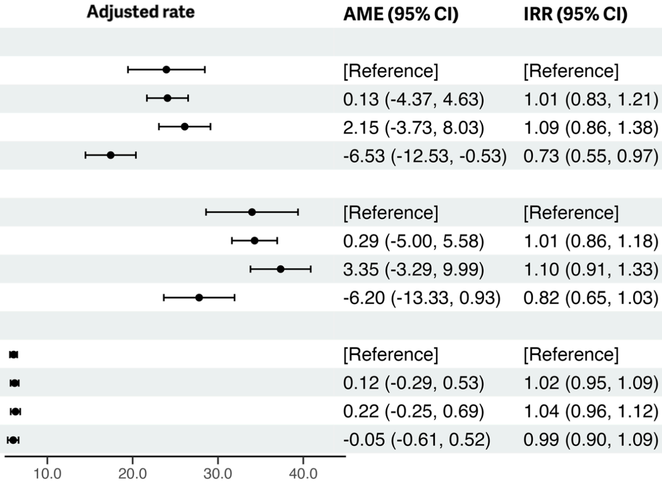

Abbreviations: AME (average marginal effect); CI (confidence interval); IRR (incident rate ratio)
Each dot represents the adjusted rate for a specific outcome at a given density level. The horizontal line extending from each dot shows the 95% confidence interval for that estimate. Models were adjusted for state-specific rates of non-White individuals, chronic hypertension, pregestational diabetes, education levels below high school, poverty rates, OBGYN/Midwives density, and year.
